# What Clinicians and Researchers Should Know About the Evolving Field of Advance Care Planning: a Narrative Review

**DOI:** 10.1007/s11606-023-08579-5

**Published:** 2024-01-02

**Authors:** Ryan D. McMahan, Susan E. Hickman, Rebecca L. Sudore

**Affiliations:** 1 Division of Geriatrics, School of Medicine, University of California, San Francisco, San Francisco, CA USA; 2 Veterans Administration Medical Center, San Francisco, CA USA; 3 Department of Community & Health Systems, Indiana University School of Nursing, Indianapolis, IN USA; 4 Indiana University Center for Aging Research, Regenstrief Institute Inc, Indianapolis, IN USA

**Keywords:** advance care planning, geriatrics, medical decision-making

## Abstract

Advance care planning (ACP) has been recognized as crucial by patients, families, and clinicians; however, different definitions and measurements have led to inconsistencies in practice and mixed evidence in the literature. This narrative review explores ACP’s evolution, innovations, and outcomes using thematic analysis to synthesize data from randomized controlled trials, reviews, and editorials. Key findings include (1) ACP has evolved over the past several decades from a sole focus on code status and advance directive (AD) forms to a continuum of care planning over the life course focused on tailored preparation for patients and surrogate decision-makers and (2) ACP measurement has evolved from traditional outcome metrics, such as AD completion, to a comprehensive outcomes framework that includes behavior change theory, systems, implementation science, and a focus on surrogate outcomes. Since the recent development of an ACP consensus definition and outcomes framework, high-quality trials have reported mainly positive outcomes for interventions, especially for surrogates, which aligns with the patient desire to relieve decision-making burden for loved ones. Additionally, measurement of “clinically meaningful” ACP information, including documented goals of care discussions, is increasingly being integrated into electronic health records (EHR), and emerging, real-time assessments and natural language processing are enhancing ACP evaluation. To make things easier for patients, families, and care teams, clinicians and researchers can use and disseminate these evolved definitions; provide patients validated, easy-to-use tools that prime patients for conversations and decrease health disparities; use easy-to-access clinician training and simple scripts for interdisciplinary team members; and document patients’ values and preferences in the medical record to capture clinically meaningful ACP so this information is available at the point of care. Future efforts should focus on efficient implementation, expanded reimbursement options, and seamless integration of EHR documentation to ensure ACP’s continued evolution to better serve patients and their care partners.

## CASE

A 75-year-old woman with metastatic cancer has been treated with surgery, chemotherapy, and radiation. She has been able to live at home alone and help care for her grandchildren. Unfortunately, while in the waiting room to see her primary care provider (PCP), she experiences acute shortness of breath and is urgently transferred to the hospital where she is admitted. What happens next depends, in part, on the advance care planning (ACP) conversations she did or did not have in the past.

### Path A

The patient had been seen by her oncologist and PCP several times over the last year. However, given her retained function, the topic of ACP had not yet been discussed. During her hospitalization, she decompensates, and the hospitalist cannot find any ACP information besides a previously documented “Full Code” when she was admitted for knee replacement surgery five years ago. The patient’s daughter is listed as an emergency contact, and the daughter instructs the team to “do everything.” The patient is transferred to the intensive care unit where she suffers a cardiac arrest shortly after intubation. She spends the next week in the ICU in pain and delirious, and she dies despite additional resuscitation efforts. The patient’s daughter is distraught and wonders if she made the right decision for her mother. The patient’s sister is distressed that she was not contacted in time to see her sister.

### Path B

Following a knee replacement surgery five years ago, the patient’s PCP helped identify a surrogate decision-maker and gave her patient-friendly ACP materials. At the time of her cancer diagnosis, her oncologist discussed how ACP could help her and her family prepare for medical decision-making and answered her questions. This allowed the patient to have several conversations with her sister and daughter. The patient had been seen by her oncologist and PCP several times over the last year where they provided support for serious illness understanding and helped her and her family cope with her cancer. They made sure to set time aside to rediscuss her ACP wishes with the primary care social worker. During her hospitalization, she decompensates, and the hospitalist goes to the ACP dashboard in the electronic health record to pull open the most recently documented discussion from the primary care social worker. It notes the patient’s sister as her decision-maker, and that if she got so sick that she might die, the most important thing would be to be surrounded by her family and “not hooked up to machines like my mom was in the end.” The hospitalist calls the sister who tearfully confirms they had spoken about this before and that she has a copy of an advance directive listing her as the durable power of attorney. The sister requests that the patient be made as comfortable as possible while she gathers the patient’s daughter and other family members to come in. The patient is placed on high-flow oxygen, the palliative care team is called, and the medical teams prioritize her comfort and supporting the family at her bedside.

## INTRODUCTION

Advance care planning (ACP) has evolved from static, statutory documents and checkboxes focused narrowly on end-of-life procedures (such as code status), to a process of preparing patients and their surrogate decision-makers for communication and medical decision-making across the life course.^1,2^ Studies demonstrate that patients and families want and expect clinicians to introduce ACP and consider ACP to be important and meaningful, especially by those who have had to make difficult medical decisions for themselves or others.^3–5^ One of the most important patient-reported ACP goals is to decrease the decision-making burden on others. Furthermore, clinicians agree that ACP is an important part of their job^6–8^ and healthcare systems, including the Centers for Medicare and Medicaid Services (CMS), acknowledge it as an important quality metric.^9^

Yet, despite this evolution in the field, wide variability in how ACP is defined and measured remains. It is also unclear what components of ACP are most effective and what clinicians can do to help support their patients given many competing priorities. The purpose of this narrative review is to discuss the evolving ACP landscape, untangle the evidence, and provide pragmatic information for clinicians and researchers.

## METHODS

Three authors (RM, SH, RS) independently searched PubMed, CINAHL, PsycINFO, Web of Science, and EMBASE to identify peer-reviewed literature published up to August 2023. We included randomized controlled trials, pragmatic implementation trials, and review articles. Additional perspective pieces and editorials by experts in the field were included. Articles were selected if the research provided insights into the evolution of ACP, highlighted innovations, or were focused on implementing tools in clinical settings with patient- or surrogate-centered outcomes. Key search terms included, but were not limited to, advance care planning, advance directives, medical and surrogate decision makers, and goal-concordant care. Data related to the objectives of this narrative review were extracted and organized into key themes. The authors discussed the interpretation and synthesis of the data through a collaborative process. The authors’ individual viewpoints and insights, based on clinical experience, research involvement, and academic contributions, facilitated the analysis. Results are presented thematically and editorialized to represent a comprehensive exploration of current practices, challenges, and opportunities in the field of ACP.

## RESULTS

### How Is Advance Care Planning Defined?

Beginning in the 1970s and after the 1990 Patient Self-Determination Act, which required hospitals to inform patients of their right to make their own healthcare decisions, the conceptualization of ACP was understood primarily as the legal-transaction process of completing advance directive (AD) forms.^10^ ADs were historically written with difficult-to-read legal language, narrowly focused on the use of life-prolonging treatments (i.e., code status and mechanical ventilation at the end of life), and infrequently used.^11–14^ Throughout the 1990s and early 2000s, ACP studies and clinical trials reflected this narrow definition and targeted varying and non-standardized outcomes. This heterogenous approach led to mixed findings. At the same time, there was a growing understanding from qualitative and other studies that patients and families need more preparation and support than a one-time form or checkbox.^4,15,16^

**Figure 1:**
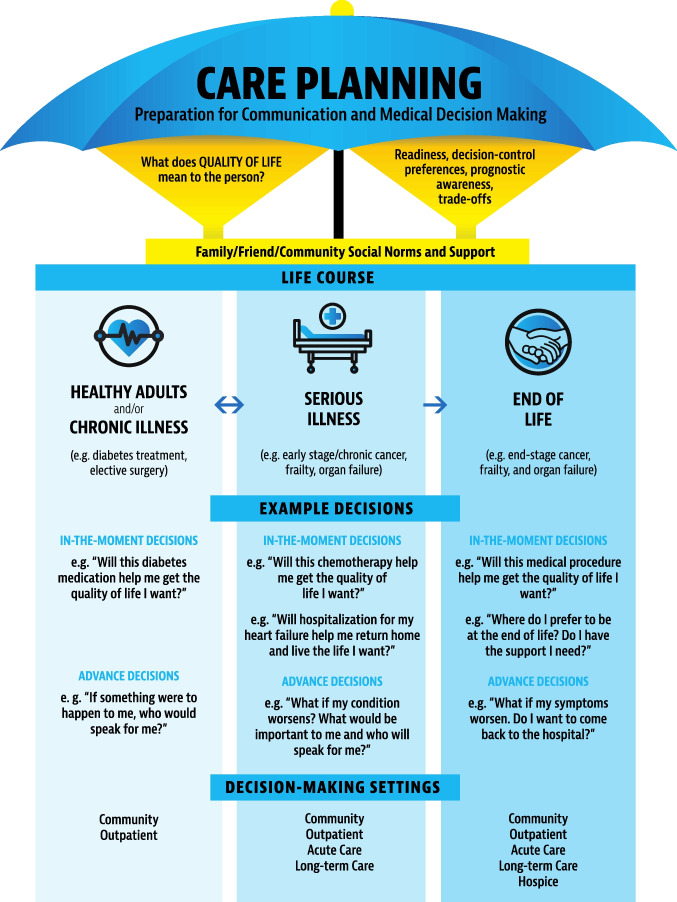
The care planning umbrella^1^. Reproduced with permission from *Journal of the American Geriatrics Society*

Based on the evolving research, investigators made a call in 2010 to expand the definition of ACP from a sole focus on code status documentation to the preparation of patients and caregivers for communication and medical decision-making.^17^ Then, in 2017, a large, international Delphi panel of over 50 clinical and legal experts defined ACP as “a process that supports adults at any age or stage of health in understanding and sharing their personal values, life goals, and preferences for current and future medical care.”^2^ Yet, the evolution continued with a growing understanding that ACP is not a simple one-time event, but a complex behavior that must be tailored to the patient’s life course; patient, surrogate, and community social norms; readiness; prognostic awareness; social support; desired control over decision-making; access to and trust in healthcare; policy; and clinical workflows.^18–21^ Therefore, in 2023, a new Care Planning framework was proposed that “reflects the updated focus on preparation for communication and medical decision-making” (for both in-the-moment and advance decisions) and “conceptualizes ACP as part of the continuum of care planning across the life course” with patients’ quality of life as the “fundamental cornerstone” (Fig. 1).^1^ Within this model, AD and POLST forms are still important and helpful for families and at the bedside, but the model reflects that they are but one piece of the ACP puzzle. ACP should reflect ongoing discussions that are tailored to the patient’s life course, include shared decision-making and illness understanding (with evolving quality of life and priorities), and prepare surrogate decision-makers.^22,23^

### How Is ACP Measured?

Early ACP research and current quality metrics often focus on AD completion, mainly because this outcome is easiest to measure. However, research has demonstrated that a sole focus on AD completion, without fostering communication or patient or surrogate preparation, has not been uniformly effective.^24,25^ Current research recognizes ACP as a complex set of behaviors, based on the behavior change model, similar to smoking cessation or exercise.^26–28^ For ACP, this includes behaviors such as identifying one’s goals for medical care, discussing goals with others over time, and if appropriate, to document preferences.^17,18,20^ Given this new level of understanding, in 2018, an international Delphi panel of experts outlined a new, more complex framework for ACP outcomes organized into five domains: *Process* outcomes included patient knowledge and perceptions of ACP, self-efficacy, and readiness, and focused on moving patients along a behavior change pathway; *Action* outcomes included concrete tasks such as discussions and/or documentation; *Quality of Care* outcomes included goal-concordant care (GCC), meaning the patient received the medical care that was aligned with their values and preferences, and overall satisfaction with communication and decision-making; *Health* outcomes included impact on health (health status and mental health); and *Health System* outcomes included healthcare utilization or cost (Table 1).^29^

**Table 1:** Advance Care Planning Outcomes Framework^29^

Outcome categories	Example outcome topics
Process	• ACP perceptions • Behavior change • Knowledge, self-efficacy, readiness • Barriers
Action	• Assign and ask a surrogate decision-maker • Discuss with family and doctors • Document in AD, POLST
Quality of care	• Satisfaction with care, communication, decision-making • Goal-concordant care
Health	• Health status • Mental health • Quality of life
Health system	• Utilization • Cost

The top-rated outcome from the Delphi panel was goal-concordant care (GCC);^29^ however, measurement of GCC is complex and there is no validated or standardized outcome to measure this construct. Furthermore, in-the-moment decisions may differ from pre-recorded choices. GCC as a concept suffers from several issues; for example, many patients lose the ability to speak for themselves and to describe their real-time goals; many people adapt to disability and to previously presumed intolerable health states; there may not be a treatment option to meet patients’ goals (e.g., cure an incurable disease); or the treatment presumed to meet a goal (such as longevity) could significantly worsen quality of life.^23,30,31^ Thus, a focus on GCC as the ultimate outcome of successful ACP, as it is currently measured (in a non-standardized way), is problematic, and attempts thus far to measure it retrospectively in the electronic health record (EHR) have resulted in mixed findings.^25,32^

With the evolving understanding that ACP is a complex, nuanced, and ongoing process, new outcomes have been developed and validated. If the goal is to measure patient or caregiver engagement in ACP and movement along the behavior change pathway (which we consider clinically significant), there are validated surveys, including a 4-item ACP readiness measure that can be used in both research and clinical settings.^33^ If the goal is to measure goal-concordant care, we recommend moving away from inherently biased retrospective chart review.^23^ Newer, prospective studies assessing real-time GCC (meaning asking patients while they are able to speak for themselves whether they are receiving the care they prefer over their life course and not just at the end of life) have shown benefit for ACP interventions, especially for interventions focused on new ACP models.^34–36^ In addition, the validated Bereaved Family Survey, developed in the Department of Veterans Affairs, asks decedents’ next of kin whether they believe the patient received goal-concordant care; and ACP has been associated with GCC using this survey.^37,38^

For quality metrics, healthcare organizations are beginning to measure a range of ACP information that can be helpful at the bedside and is considered clinically meaningful ACP. “Clinically meaningful ACP” not only includes AD forms and orders, such as POLST or code status, but also includes documented oral ADs and narrative notes or information in problem lists. Historically, a significant portion of clinically meaningful ACP has been buried in the notes and hard to find at the point of care.^39^ However, EHR dashboards for ACP information and the use of “smart phrases” as well as natural language processing and machine learning algorithms are helping to bridge this gap so that clinically meaningful information can be available for both research and clinical purposes.^40,41^

### Is ACP Effective?

Multiple ACP reviews have been conducted over the years, including recent scoping^25,42^ and narrative reviews,^19^ in addition to a 2018 systematic review of 80 systematic reviews of ACP studies.^32^ This review highlighted mixed results for several ACP outcomes and identified numerous limitations in the existing research, including the low-quality mixed methods evidence, leading some to question the efficacy of ACP.^43^ A consistent thread throughout the multiple reviews has been the acknowledgement that ACP is desired by patients and surrogates; yet, there is a need for future research to standardize definitions and outcomes and to focus on implementation. The 2021 scoping review included only high-quality randomized trials since the publication of updated ACP definition in 2010, used the Delphi-developed ACP Outcomes Framework,^29^ and found that the results for all interventions and outcomes were largely positive.^25^

For interventions, patient-facing written, multi-media, and facilitated discussion intervention trials were most consistently positive (> 70%), whereas just over half of the clinician training interventions were successful. For outcomes, positive results were seen for ACP studies that addressed *process* outcomes (e.g., knowledge, readiness) and *action* outcomes (e.g., communication, documentation). *Quality of Care* outcomes were generally positive, including patient and caregiver satisfaction with medical care, decision-making, and communication, which is important given the desire for value-based care and the growing significance of patient satisfaction surveys. However, GCC rarely showed positive results and used retrospective and non-standardized measures. *Health* outcomes for patient quality of life (which may not be expected to change in the case of serious or terminal illness) and healthcare utilization (which is not considered to be a patient-centered outcome) were mixed. The most striking finding was that in almost all cases, ACP decreased surrogate anxiety, depression, PTSD, complicated grief, and caregiver burden, and in the one study it was measured, also decreased clinician moral distress. This means that ACP is meeting patient stated goals for ACP, which is to reduce decision-making burden on their loved ones. Indeed, despite some discussion about the efficacy of all aspects of ACP, the field uniformly agrees on the importance of surrogate preparation.^19,23,44^

### What ACP Tools Can Clinicians Use to Support Patients?

There are several resources for clinicians, from all interdisciplinary backgrounds, to start ACP discussions in an efficient way.^19,45,46^ These include easy-to-use, step-by-step scripts for clinicians,^17,47^ as well as online and other trainings.^21,47–52^ We recommend beginning by asking all patients: (1) if they have identified a surrogate decision-maker whom they trust to make decisions in the event they are unable to make decisions for themselves; and (2) what they have already discussed with their surrogate in terms of their role and current medical preferences (Table 2). This provides helpful information including whether the individual is socially isolated (and needs additional help focusing on AD completion and other planning) or whether the person has begun to prepare their surrogate decision-maker through discussions about their goals, values, preferences, and what quality of life means to them. Based on these responses, a tailored approach can then be made based on patients’ readiness and life course (Fig. 1)^1^ and using the aforementioned communication guides. Working with interdisciplinary team members can also provide valuable assistance. This step-by-step, team-based approach to ACP can help make the process less daunting and time consuming for any one individual team member in busy outpatient practices.

**Table 2:** Example ACP Wording and Phrasing for Clinicians*

Educate and normalize ACP	I wanted to take a moment to talk about advance care planning. This involves choosing an emergency contact and discussing the medical care that is important to you
Choose a surrogate decision maker	Is there anyone you trust to make medical decisions for you if there ever came a time when you could not speak for yourself? Have you asked this person to play this role? What have you talked about?
Decide what matters most in life	Have you ever completed an advance directive? This is a legal form that lets you write down the name of your advocate or medical decision maker and your preferences for medical care If Yes: Do you remember what you wrote down? Do you still feel the same way? Do you know where this form is? How do you define good quality of life? What brings your life meaning and joy? What are you most looking forward to? What do you most worry about? Have you seen someone on TV or had someone close to you who had serious illness? What went well and what did not go well? Why? Are there any health conditions that would be unacceptable or very hard on your quality of life?
Discuss leeway in surrogate decision making	Some people want their decision maker to follow their wishes exactly. Others give their decision maker flexibility or leeway to work with the medical team to make decisions that are in the sick person’s best interest at that time Is it OK to use your medical wishes as a general guide and to change your decisions if your doctors and care team think it is best at that time? Or, are there some decisions you never want changed even if the doctors are recommending it?
Connect patients’ view of quality of life to treatment decisions	Based on what you told me about what brings your life meaning/how you feel about your loved one’s experiences/how you felt about your last hospitalization, it sounds as though (e.g., this treatment option, etc.) may be something that you would/would not want for yourself. Is this correct?
Refer patients to evidence-based programs	I have some easy-to-read materials that will help you to make medical decisions I’d like you to review these materials before our next visit. (Example: The PREPAREforYourCare.org website includes easy-to-read pamphlets and legal advance directive forms for all US states in multiple language that can be printed and handed to patients)

^*^Phrases adapted from Sudore, *Annals of Internal Medicine* 2010^17^and clinician materials provided on www.PREPAREforYourCare.org^47^

While documentation of a surrogate decision-maker is important for any adult, as people develop serious illness, frailty, or are near the end of life, documentation of treatment preferences and values is also important. To help prepare and “prime” patients for these conversations, we recommend providing evidence-based ACP tools prior to a medical visit.^45,46,48,51–63^ For example, the step-by-step, easy-to-read PREPARE for Your Care online ACP program, that was developed with and for English- and Spanish-speaking older adults, surrogates, and the community, has been shown in randomized trials to significantly increase ACP documentation in the medical record.^64–66^ The program also primes patients and resulted in approximately 50% greater patient empowerment to initiate ACP discussions during primary care visits and to report a 50% increase in real-time goal-concordant care.^35,67,68^ CMS allows for billing of ACP but requires 16 minutes of conversation;^69^ which is hard in a busy primary care practices. However, it is permissible to have billing providers begin the conversation then hand further discussion to another qualified healthcare professional so that ACP can be a team-based process. Group medical visits are also an efficient way to reach several patients at once and have been shown to be highly effective.^70–72^

To facilitate clinically meaningful ACP, all discussions should be documented so that they can be part of the EHR. As documentation options differ by institution, it is imperative for clinicians to learn where and how to document ACP in their EHR so that it can be found when needed at the point of care. For example, does your institution have a central ACP tab or dashboard where all ACP documentation can be found? If so, it is important to learn what ACP note type, problem list, or EHR smart phrase will automatically populate the centralized ACP dashboard so that your hard work and important clinical information does not get lost in the shuffle. If not, this is a good time to advocate for a centralized location in the EHR for ACP at your institution as several of the leading EHR companies have already created this functionality.

For advance directive documentation, we advocate using ADs that promote universal access by adhering to health literacy and patient preferred language principles to decrease disparities. In addition, we recommend ADs not only include information about end-of-life treatment preferences, but also include questions about patient values and quality of life.^53,54,73^ It is not only the checkbox decision that is important, but the “why” behind that decision that helps when clinicians and surrogate decision-makers are wrestling with what to do at the bedside for a myriad of potential decisions. The PREPARE for Your Care easy-to-read, language-appropriate ADs that focus on values and quality of life have been shown in randomized trials to decrease health disparities in ACP.^64,65,73^ They are free to the public in all US states and are available in multiple languages.^47^

Finally, for those patients at the end of life with stable preferences to limit interventions, we recommend the use of POLST, which is known by other names such as Physician Orders for Scope of Treatment or POST and Medical Orders for Life Sustaining Treatment or MOLST. These portable medical orders are actionable and transferable inside, between, and outside different healthcare settings such as the hospital to nursing home. Research suggests POLST orders help support goal-concordant care.^74^ Our team has developed a free video to help explain POLST to patients and is available, along with other resources, on the National POLST website (www.POLST.org).

### What Is the Future of ACP?

In some ways, ACP research is still in its adolescence. There is much left to learn as the field is beginning to unpack the complexity of ACP, including the impact of policy, quality metrics, EHR frameworks, clinical workflows, and the influence of social norms and community support. One initial hope for ACP was that this “simple” task would somehow solve the problems of the harmful overutilization at the end of life in our complex US health system. However, ACP is one piece in a much larger, complex, and entrenched clinical-industrial puzzle. Without a systems and holistic approach, it is unreasonable to expect ACP alone to turn the tide on healthcare utilization. Application of systems-level methodologies, such as human factors engineering and implementation science, are needed to help us further unpack how to do ACP in the most tailored, efficient, and effective way.^1^

We are heartened by the increase in community-initiated ACP programs in senior centers, religious centers, and other community organizations.^75–78^ In health systems and the community, we are also encouraged by the impact of lay health navigators to introduce ACP.^79,80^ Furthermore, we are hopeful that ACP billing may expand to other care team members, such as social workers, who are exceptionally trained and skilled at these discussions.^39,81–83^ When measuring ACP, more appropriate short- and long-term outcomes of ACP should be focused on patient and surrogate satisfaction and decreased surrogate and clinician decision-making burden and moral distress. Finally, for measurement of ACP and quality metrics, a focus on “clinically meaningful ACP” (narrative notes in addition to forms and orders), is also important. With the increased use of natural language processing, machine learning, and generative artificial intelligence programs (i.e., ChatGPT), as well as state and regional health information exchanges and networked EHR systems, we hope that documenting and accessing ACP information to support patient care will one day be seamless, wherever the patient is receiving care.^84^

## CONCLUSION

ACP has evolved over the past several decades from a sole focus on code status and AD forms to a continuum of care planning over the life course focused on tailored preparation for patients and surrogate decision-makers. To make things easier for patients, families, and care teams, clinicians and researchers can use and disseminate these updated and evolved definitions; provide patients validated, easy-to-use tools that prime patients for ACP conversations and decrease health disparities; use easy-to-access clinician training and simple scripts for interdisciplinary team members; and document patients’ values and wishes in the medical record to capture clinically meaningful ACP so this information is available at the point of care. There is still significant systems and policy-based work needed to make ACP more efficient and effective, but it is clear that patients and surrogates want, need, and deserve better preparation. There are also growing resources, interventions, and systems-level changes that will continue to improve this process over time. ACP continues to evolve. Stay tuned.
